# Effects of Clonal Integration on Microbial Community Composition and Processes in the Rhizosphere of the Stoloniferous Herb *Glechoma longituba* (Nakai) Kuprian

**DOI:** 10.1371/journal.pone.0108259

**Published:** 2014-09-22

**Authors:** Ningfei Lei, Jun Li, Shijun Ni, Jinsong Chen

**Affiliations:** 1 Chengdu University of Technology, Chengdu, China; 2 Sichuan Normal University, Chengdu, China; Beijing Forestry University, China

## Abstract

The effects of rhizodeposition on soil C and N availabilities lead to substantial changes of microbial community composition and processes in the rhizosphere of plants. Under heterogeneous light, photosynthates can be translocated or shared between exposed and shaded ramets by clonal integration. Clonal integration may enhance the rhizodeposition of the shaded ramets, which further influences nutrient recycling in their rhizosphere. To test the hypothesis, we conducted a pot experiment by the stoloniferous herb *Glechoma longituba* subjected to heterogeneous light. Microbial biomass and community composition in the rhizosphere of shaded offspring ramets, assessed by phospholipid fatty acids (PLFAs) analysis, were markedly altered by clonal integration. Clonal integration positively affected C, N availabilities, invertase and urease activities, N mineralization (*N_min_*) and nitrification rates (*N_nitri_*) in the rhizosphere of shaded offspring ramets. However, an opposite pattern was also observed in phenoloxidase (POXase) and peroxidase (PODase) activities. Our results demonstrated that clonal integration facilitated N assimilation and uptake in the rhizosphere of shaded offspring ramets. The experiment provides insights into the mechanism of nutrient recycling mediated by clonal integration.

## Introduction

Rhizosphere, a zone of usually high microbial turnover and activity, has been coined to describe the soil adjacent to and influenced by plant roots [1]. Plant-derived root exudates are primary sources of labile C inputting to soil [2], [3]. These labile C sources rapidly metabolized by microorganisms may generally stimulate their growth or succession in the rhizosphere [4], [5]. So, plant roots exert strong influences on the rhizosphere through ‘rhizodeposition’ (root exudation such as sugars, amino acids, organic acids and hormones, as well as mucilage, enzymes, sloughed root cells and C allocated to root-associated symbionts) [6].

In the form of rhizodeposition, photosynthates released into soil by plant roots are a major source of carbon, energy or structural material for soil microorganisms and affect the microbial community composition in the rhizosphere [7]–[11]. Fungi, especially ectomycorrhizal (ECM) fungi involved in nitrogen turnover (e.g. mineralization and nitrification), prefer the substrates with larger C/N ratios [12]. Microbial processes, such as extracellular enzyme activities [1], [13], [14], N mineralization and nitrification [15], [16], are mediated by specific groups of microorganisms in the rhizosphere. Two experiments to disrupt root exudation into the soil demonstrated that decreased resource availability negatively affected nitrogen mineralization and nitrification in the rhizosphere via rhizodeposition from plant root [1], [15]. So, microbial processes are highly sensitive to the availabilities of labile C and N in the rhizosphere [5].

Clonal plants can translocate or share resources, such as carbohydrates, water and nutrients among interconnected ramets through clonal integration [17]. Shading may have negative effects on photosynthetic capacity and growth performance of plants [18], [19]. Clonal integration may alter resource levels of ramets under heterogeneous habitats [20], [21]. So, enhanced photosynthates availability caused by clonal integration may have a significant influence on microbial community composition and processes in the rhizosphere of the ramets subjected to low light availability stress. Further, microorganisms present in the rhizosphere may mediate nutrient availability for plants by carrying out a wide spectrum of decomposition processes. As mentioned-above, rhizosphere processes may play a vital role in community or ecosystem nutrient cycling [1]. However, studies on the mechanism of nutrient recycling mediated by clonal integration are rare in the rhizosphere. A pot experiment was conducted by the stoloniferous herb *Glechoma longituba* subjected to heterogeneous light (mother ramets suffering from full sun versus offspring ones suffering from 80% shade). Comparing with severed offspring ramets, we predicted that connected offspring ramets displayed (1) higher C and N availabilities in the rhizosphere. Based on effects of C and N availabilities on microbial community composition and processes, we expected that connected offspring ramets exhibited (2) higher microbial biomass and different microbial community composition in the rhizosphere; (3) higher extracellular enzymes activities in the rhizosphere; (4) greater N mineralization and nitrification rates in the rhizosphere.

## Material and Methods

### Plant species and experimental design

*G. longituba* (Lamiaceae) is a stoloniferous perennial herb. Its monopodial stolons are able to creep on the ground. Ramets can develop on all stolon nodes. A genet or fragment consists of a number of ramets connected by stolons for a certain period of time. Each ramet has two zygomorphic single leaves originating from a stolon node. Every leaf axil bears one bud that may grow into a secondary stolon. The plant is generally found in forests, on roadsides or by creeks and distributed all over China except for the Northwest [22].

In May 2012, ten original clonal fragments of *G. longituba* were collected from a forest understory in Suining City (30°10′∼31°10′N; 105°03′∼106°59′E), Sichuan Province, China. The sampling site did not belong to the part of any farms or national parks. *G. longituba* is widespread in China and not an endangered or protected species, so we did not need any relevant permissions/permits for plant sample collection. These original plants were at least 100 m apart from one another. They were propagated in a greenhouse with a mean temperature of 22±8°C. The plants were watered and fertilized as needed.

**Figure 1:**
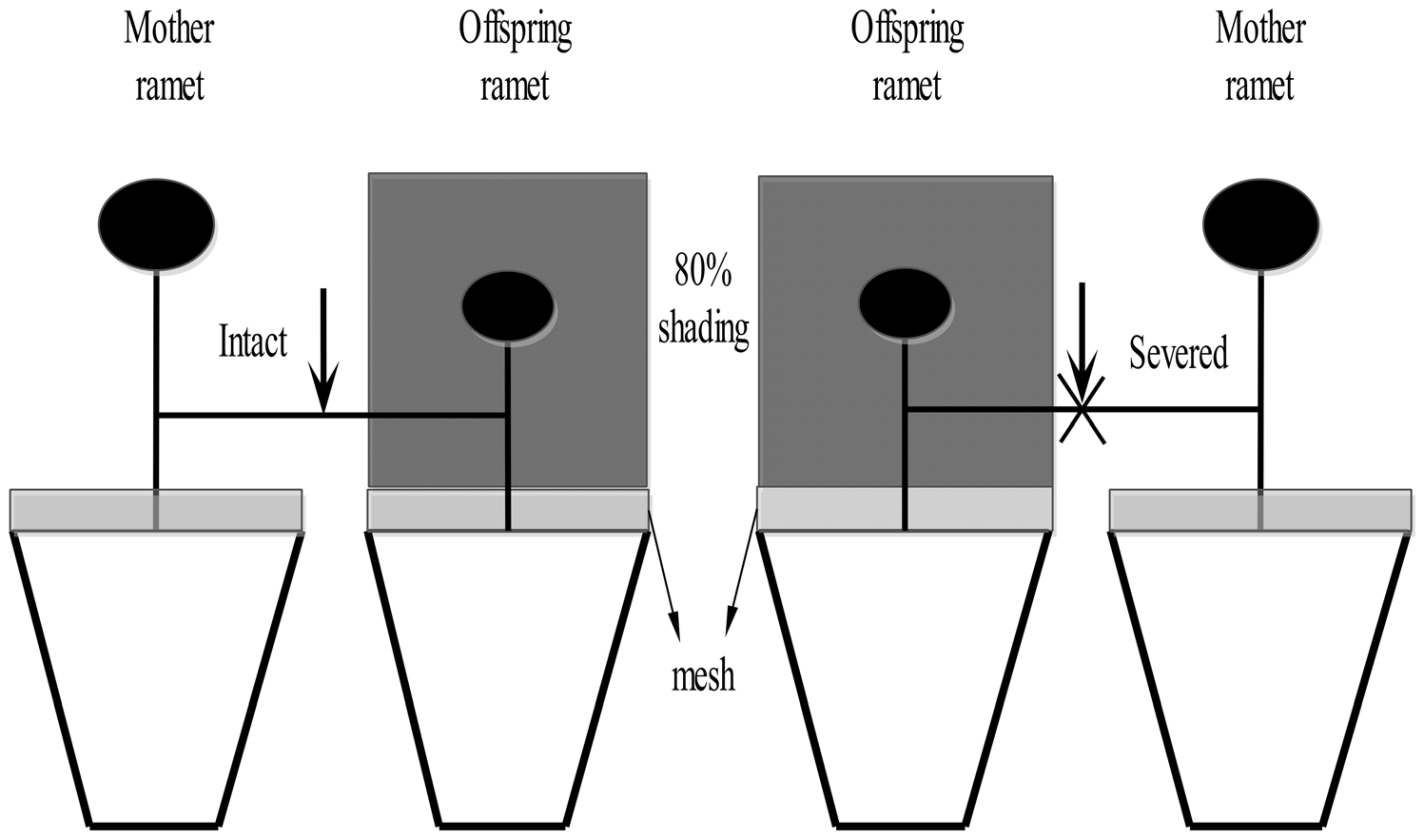
Schematic diagram of the experimental design. The clonal fragment consists of mother ramet and its successional offspring ramet. The offspring ramets were subjected to the 80% shading treatment, and the mother ramets were exposed to full sun. The stolon between mother and offspring ramets remained intact or severed. The mesh was covered on the top of each pot to avoid potential effects of litters.

In June 2013, each clonal fragment consisting of a mother and an offspring ramet with similar size was selected. The two ramets were planted separately in two adjacent plastic pots (10 cm in diameter, 8.5 cm in height) filled with a 3∶1 mixture of humus soil and sand. Plants were watered regularly with distilled water to prevent water stress. After two weeks of growth, offspring ramets were subjected to a 80% shading treatment and the other mother ramets were grown in full sun, whereas stolons between the mother and the offspring ramets were either severed or remained intact (Fig. 1). No new offspring ramets were produced during the two weeks of recovery. Shading was imposed by placing small shade cages covered with black cloth above the pots. The mesh was covered on the top of each pot to avoid potential effects of litters. Only original ramets were allowed to root during the experiment. Each treatment was replicated 10 times and all treatments included clonal fragments from the 10 original plants. All replicates of each treatment were randomly located on benches in a greenhouse. Because soil closely adhering to the roots (up to 2 mm around the root) was considered as rhizosphere soil, the experimental procedure was repeated 10 times to collect enough soil sample. The experiment lasted for 10 weeks.

### Soil sampling and assays

At the end of experiment, rhizosphere soil was sampled according to the shaking root method [23]. The rhizosphere soil of shaded offspring ramets was separated from roots by hand, sieved (<2 mm mesh) and stored at −20°C.

Soil microbial biomass carbon (*C_mic_*) and microbial biomass nitrogen (*N_mic_*) were analyzed using chloroform fumigation-extraction (CFE) method [24], [25]. Briefly, 20 g of fresh, sieved soil was used for the fumigation and non-fumigation treatments, both extracted using 0.5 M K_2_SO_4_ at a ratio of 1∶4 (w/v), shaken for 30 min and filtrated through a Whatman no. 42 filter paper. The K_2_SO_4_-extract of both fumigated and non-fumigated samples were analyzed immediately for dissolved organic carbon (DOC) and dissolved organic nitrogen (DON) using a TOC/TN analyzer (elementar vario TOC SELECT, Germany). *C_mic_* and *N_mic_* were calculated using the following equations: *C_mic_* (or *N_mic_*) = 2.22×*E_B_* [26], where *E_B_* was the difference of carbon (or nitrogen) extracted from fumigated soil between non-fumigated soil. Soil moisture was detected gravimetrically, i.e. a sample of 20 g was oven-dried at 105°Cfor 48 h until a constant weight. Total soil organic carbon (TOC) and total nitrogen (TN) were determined using an elemental analyzer (elementar vario MACRO CUBE, Germany). Soil pH was measured in a ratio of 1: 2.5 (soil: water, w/v).

### N mineralization and nitrification

N mineralization and nitrification were assessed by the modified anaerobic incubation method [27]. Briefly, fresh soil samples (5 g) were placed into a 200 mL plastic bottles, 10 mL deionized water was added to the bottles to thoroughly submerge the soil. The plastic bottles were sealed with stopper to avoid water evaporation during incubation, and placed in a constant temperature (40°C) incubator for 7 days. At the beginning of the incubation experiment, pre-incubation soil was sampled to measure the initial concentrations of NH_4_^+^-N and NO_3_^−^-N. After a week of incubation, the post-incubation soil samples were mixed with 40 mL of 2 M KCl using a 1: 8 soil: extractant (w/v) ratio, shaken for 30 min on a reciprocal shaker; then the extracts were filtered through prewashed Whatman no. 42 filter papers and supernatants were stored at −20°C until analysis of NH_4_^+^-N and NO_3_^−^-N concentrations. The NH_4_^+^-N and NO_3_^−^-N concentrations were separately measured by spectrophotometry using the ammonium indophenol blue method and the cadmium reduction method [28]. All concentrations of NH_4_^+^-N and NO_3_^−^-N were based on dry soil weight and expressed on a mg·g^−1^ (*DW*). The N mineralization rate (*N_min_*) was calculated as the changes in the inorganic N (NH_4_^+^-N, NO_3_^−^-N) content from time zero to 7 days. A similar formula was used to calculate N nitrification rate (*N_nitri_*) [29], [30]:



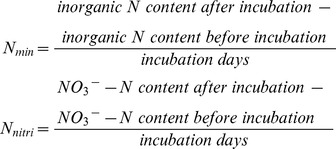



### Extracellular enzymes activities assays

Invertase activity was measured by the modified method [31]. Briefly, fresh soil (1.0 g) was added to 5 mL 0.1 M saccharose in 0.1 M Na-acetate buffer (pH 4.65) in a 50 mL reaction flask and incubated for 1 h at 30°C. After of incubation, the mixture was transferred immediately to a freezer for 10 min to stop the enzymatic reaction, centrifuged at 3500 *g* for 10 min and the reaction products were determined in the supernatants. The concentrations of glucose produced by saccharose hydrolysis were determined by the Nelson-Somoji reagent at 660 nm [32]. Invertase activity was expressed in µg glucose g^−1^ *DW* h^−1^.

For the determination of urease activity, the modified procedure was adopted according to the description [33]. Briefly, 5 g fresh soil was incubated with 2.5 mL 0.08 M urea solution and 20 mL borate buffer (pH 10.0) for 2 h at 37°C. Released ammonium was extracted using 50 mL 2 M KCl solution, and determined colorimetrically at 690 nm.

POXase activity was measured according to an improved procedure [34]. Briefly, 1.0 g of fresh soil was added to 3 mL of reagent solution (obtained by mixing 1.5 mL of catechol solution with 1.5 mL of proline solution) and 2 mL of phosphate buffer (0.1 M, pH 6.5). The suspension was swirled and incubated at 37°C for 1 h, then reaction was stopped by cooling and adding 5 mL of ethanol. The mixture was centrifuged at 4000 *g* at 4°C for 5 min. The absorbance of the supernatant fraction was determined at 525 nm. Assays without soil and catechol were carried out simultaneously as controls. POXase activity was expressed as µmol oxidized catechol (*o-*catechol) g^−1^ *DW* h^−1^.

PODase activity was determined with 3,3′,5,5′-tetramethylbenzidine (TMB) as the substrates [35]. Samples of fresh soil (4 g) were mixed with 200 mL cold acetate buffer (5°C, 50 mM, pH 5.0) on a vortex mixer for 30 s at high speed. The soil suspension was then diluted 20-fold in acetate buffer. Aliquots of 0.25 mL were transferred to 2 mL centrifuge tubes. 0.5 mL preheated TMB reagent (25°C) was added to the centrifuge tubes and the tubes were incubated in a constant temperature (25°C) incubator. After 2 h, peroxidase reaction was terminated by adding 1.2 mL sulfuric acid (0.2 M). The tubes were then centrifuged under dark and absorbance of the supernatants were read at 450 nm. Controls were performed using acetate buffer substituted TMB to confirm that there was no photo-oxidation of TMB.

### Microbial community composition

Microbial community composition was assessed using phospholipid fatty acids (PLFAs) [1]. (1) Extraction: A sample of 8.0 g fresh soil was extracted with a mixture of citrate buffer (0.15 M, pH 4.0 with NaOH), chloroform and methanol at a ratio of 0.8: 1: 2 (v/v/v). Suspension was shaken darkly at 25°C for 30 min, centrifuged at 10000 *g* for 0.5 h and the supernatant was transferred to new vials. Chloroform and citrate buffer were added to supernatant for separation of the phases. After 18 h, the organic phases were removed and dried under a stream of dry N_2_. (2) Chromatography: lipids were redissolved in chloroform and neutral lipids were separated from phospholipids on silica columns by elution with chloroform (5 mL), acetone (10 mL) and methanol (5 mL) gradually. Methanol-phase was collected and dried under N_2_ stream. (3) Methyl esterification: phospholipids were subsequently converted to fatty acids methyl esters (FAMEs) by alkaline methanolysis. Phospholipids were dissolved in 1 mL of methanolic 0.2 M KOH and 1 mL of methanol-toluene (1∶1, v/v) and incubated for 15 min at 37°C, then mixed with 2 mL of deionized water, 0.3 mL of acetic acids (0.2 M) and 2 mL of hexane, swirled and centrifuged for 10 min. The hexane-phase was removed and dried under N_2_ stream. After adding 100 µL of a solution of methyl-nonadecanoate (C19:0, 25 ng µL^−1^) as an internal standard, FAMEs were dissolved in C19:0 and analyzed by capillary gas chromatography (Agilent Technologies, 6850N-GC System, USA). Concentration of single FAMEs was calculated using the internal standard (C19:0) peak as a reference according to the following formula:



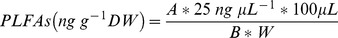



Where A and B were the peak areas of each fatty acid methyl ester and internal standard, respectively; W was the oven-dry soil weight (*DW*).

PLFAs used as biomarkers for specific groups of soil microorganisms and were designated according to an standard nomenclature: ((*a,i,cy*)*X: YωZ(c,t)*), where the *X* referred to the number of C atoms, the *Y* indicated the number of double bonds followed by the position (*ω*) and distance (*Z*) of the double bonds from the methyl end. The prefixes *a*, *i* indicated *anteiso*- and *iso*-branching; the suffixes *c*, *t* referred to *cis*- and *trans*-double bonds; *cy* represented cyclopropyl-group. The fatty acids used as biomarkers for specific groups of soil organisms were listed in Table 1.

**Table 1:** PLFAs biomarkers of different microbial groups were used in the study.

Soil microbial groups	PLFAs biomarkers	References
Bacteria	i13:0, i14:0, 14:0, 15:0, 16:0, 17:0, i15:0, i16:0, i17:0, a15:0, 16:1ω7c, a17:0, 18:0, cy17:0, cy19:0, 16:1ω7c, cy19:0ω9c, 10Me16:0	[13], [43], [63]–[65]
Actinomycete	10Me18:0	[65]
Fungi	18:1ω9, 18:2ω6,9	[13]
Ectomycorrhizal fungi	18:2ω6,9	[12]
Gram-positive bacteria	i13:0, i14:0, 14:0, i15:0, i16:0, i17:0, a15:0, a17:0, 10Me16:0	[13], [64]
Gram-negative bacteria	18:0, cy17:0, cy19:0, 16:1ω7c, cy19:0ω9c	[43], [64]
Bacteria/Fungi	(i13:0, i14:0, 14:0, 15:0, 16:0, 17:0, i15:0, i16:0, i17:0, a15:0, 16:1ω7c, a17:0, 18:0, cy17:0, cy19:0, 16:1ω7c, cy19:0ω9c, 10Me16:0)/(18:1ω9, 18:2ω6,9)	

### Statistical analysis

Soil pH, moisture, C and N availabilities, microbial biomass, extracellular enzymes activities, N mineralization and nitrification rates in the rhizosphere of shaded offspring ramets were investigated by one-way ANOVA. Microbial community composition of shaded offspring ramets was analyzed by a principal component analysis (PCA) using specific PLFAs biomarkers. For PCA analysis, scores of different soil microbial groups were expressed as percentage of the total PLFAs in the sample. Pearson correlations were used for relating PLFAs concentrations of different microbial groups to extracellular enzymes activities, N mineralization and nitrification, C and N availabilities. Significance was set at *p* = 0.05 level. If needed, data were natural logarithm-transformed or arcsine-transformed in order to achieve normality and homogeneity of variance. All statistical analyses were performed using SPSS 20.0 software (SPSS, Chicago, IL, USA).

## Results

### Changes in soil properties

Clonal integration significantly increased TOC, DOC and DON concentrations in the rhizosphere of shaded offspring ramets as well as C/N, whereas no effects of clonal integration on soil moisture, pH and TN were observed in the rhizosphere of shaded offspring ramets (Table 2). *C_mic_* and *N_mic_* were significantly higher in the rhizosphere of shaded, connected offspring ramets compared to shaded, severed offspring ramets (Table 2). Concentrations of inorganic nitrogen (NH_4_^+^-N and NO_3_^−^-N) in the rhizosphere of shaded offspring ramets were also markedly increased by clonal integration. Meanwhile, NH_4_^+^-N concentration was evidently higher than NO_3_^−^-N concentration, regardless of stolon connection or severing (Table 2).

**Table 2:** Effects of clonal integration on soil properties in the rhizosphere of shaded offspring ramets.

Soil properties	Treatments	
	Connected	Severed
TOC (g·kg^−1^)	18.42±0.19	17.87±0.06 **
TN (g·kg^−1^)	1.96±0.03	2.04±0.08^ns^
TOC/TN (C/N)	9.42±0.22	8.75±0.37 *
DOC (mg·g^−1^)	1.06±0.038	0.96±0.04 **
DON (mg·g^−1^)	0.58±0.037	0.52±0.006 **
*C_mic_* (µg C·g^−1^)	403.55±23.23	292.17±40.28 ***
*N_mic_* (µg N·g^−1^)	351.57±20.21	74.60±20.19 ***
soil moisture (%)	16.49±2.35	17.91±1.37^ns^
pH (soil: water = 1: 5)	7.75±0.07	7.47±0.15^ns^
NH_4_^+^-N (mg·kg^−1^)	35.67±5.14	22.54±2.46 *
NO_3_^−^-N (mg·kg^−1^)	13.40±0.16	6.61±0.27 ***

Values are means ± SE (standard errors). The significant differences between connected offspring and severed offspring ramets were indicated by *** (*p*<0.001), ** (*p*<0.01), * (*p*<0.05) and ns (not significant); n = 10. Abbreviations: TOC, total organic carbon; TN, total nitrogen; DOC, dissolved organic carbon; DON, dissolved organic nitrogen; *C_mic_*, microbial biomass carbon; *N_mic_*, microbial biomass nitrogen.

### Changes in microbial community composition

**Figure 2:**
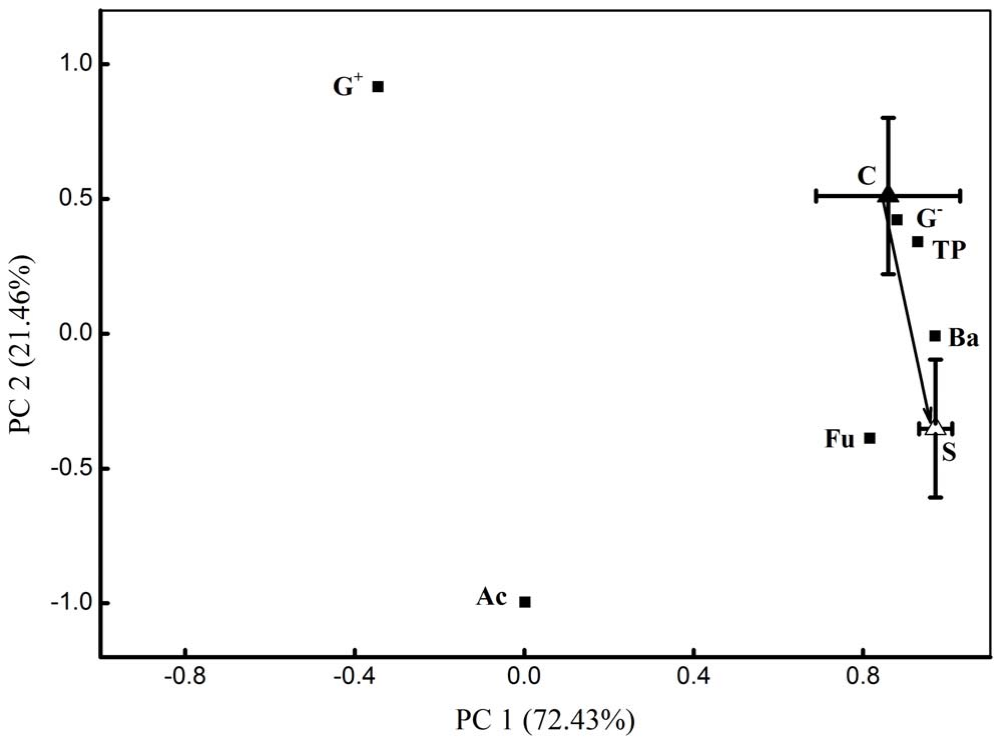
Microbial community composition described by a principal component analysis of the concentrations of PLFAs. Error bars showed standard error of the mean of PCA weighted loading values for connected (C) and severed (S) shaded offspring ramets. Black squares represented PCA weighted loading values of microorganisms. Microbial groups abbreviations: TP, total PLFAs; Ba, bacteria; Fu, fungi; Ac, actinomycete; G^+^, gram-positive bacteria; G^−^, gram-negative bacteria, n = 10.

**Figure 3:**
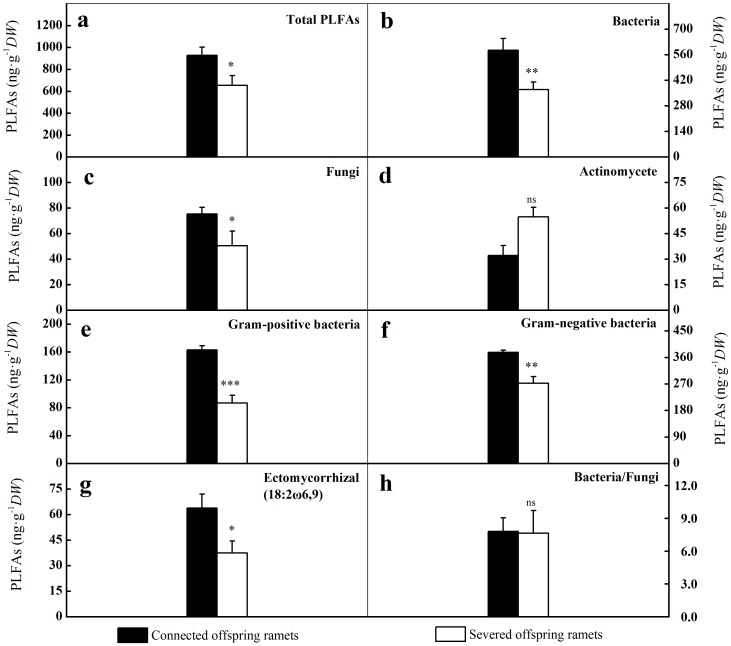
Concentrations or ratios of soil microbial groups PLFAs in the rhizosphere of shaded offspring ramets. Significant differences between connected offspring ramets (black bars) and severed offspring ones (open bars) were indicated by *** (*p*<0.001), ** (*p*<0.01), * (*p*<0.05) and ns (not significant). Error bars represented standard errors, n = 10.

A principal component analysis (PCA) based on PLFAs biomarkers of different microbial groups revealed that microbial community composition was clearly distinct in the rhizosphere of shaded, connected offspring ramets compared to shaded, severed offspring ramets (Fig. 2). The results of PCA were further supported by the absolute PLFAs concentrations for different microbial groups (Fig. 3). The PLFAs concentrations of bacteria, fungi, Gram-positive bacteria and Gram-negative bacteria were significantly increased by clonal integration as well as the total PLFAs concentration (Fig. 3a,b,c,e,f). The biomarker (18:2ω6,9) accounted for 86% of total fungal PLFAs concentrations in the rhizosphere of shaded, connected offspring ramets, the proportion was slightly decreased (75%) in shaded, severed offspring ones. Specially, clonal integration distinctly increased the PLFAs concentration of ECM fungi (18:2ω6,9) by 72% (Fig. 3g). However, effects of clonal integration on the PLFAs concentration of actinomycete and Ba/Fu were not observed (Fig. 3d, h).

### Changes in microbial processes

**Figure 4:**
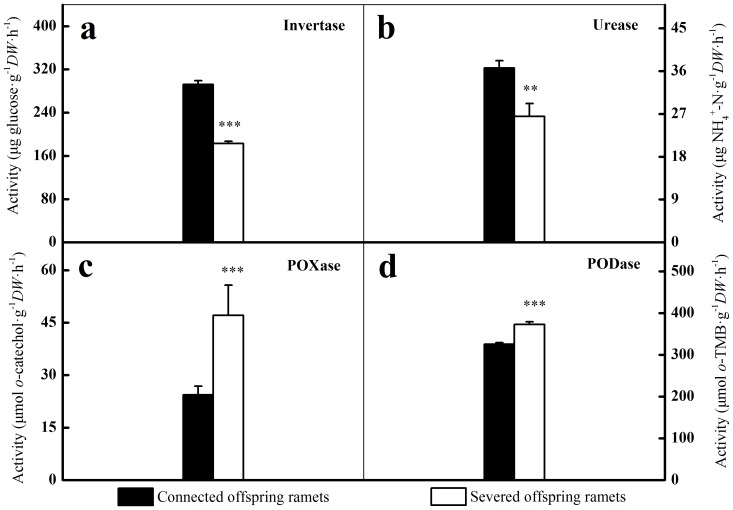
Extracellular enzymes activities involved in the depolymerization of C, N from SOM. Invertase (a), Urease (b), POXase (c) and PODase (d) were measured in the rhizosphere of shaded offspring ramets. Significant differences between connected offspring ramets (black bars) and severed offspring ones (open bars) were indicated by *** (*p*<0.001) and ** (*p*<0.01). Error bars represented standard errors, n = 10.

**Figure 5:**
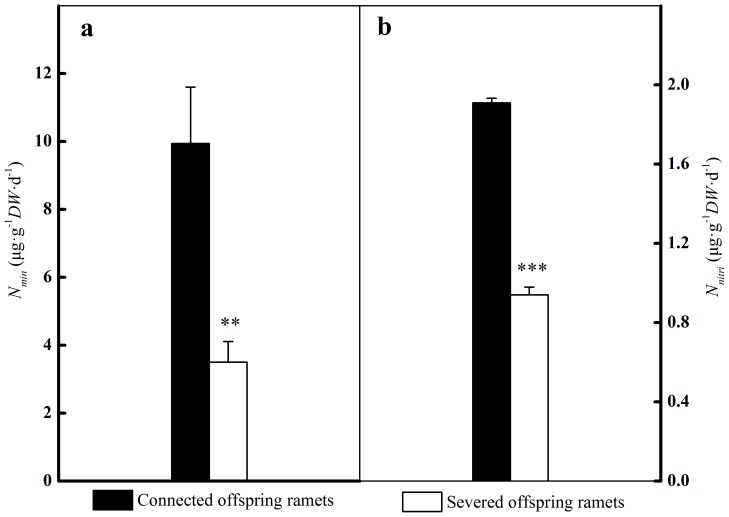
N mineralization rate (a) and nitrification rate (b) in the rhizosphere of shaded offspring ramets were measured by anaerobic incubation methods. Significant differences between connected offspring ramets (black bars) and severed offspring ones (open bars) were indicated by *** (*p*<0.001), ** (*p*<0.01). Error bars represented standard errors, n = 10.

Compared to shaded, severed offspring ramets, invertase and urease activities in the rhizosphere of shaded, connected offspring ramets were increased by 59.8% (*p*<0.001) and 38.9% (*p* = 0.005) respectively (Fig. 4a,b). On the contrary, POXase and PODase activities in the rhizosphere of shaded, connected offspring ramets were decreased by 47.8% (*p*<0.001) and 12.7% (*p*<0.001) respectively (Fig. 4c,d). *N_nitri_* accounted for only 17% of *N_min_* in the rhizosphere of shaded, connected offspring ramets, compared to 28% in the rhizosphere of shaded, severed offspring ones (*p* = 0.034). *N_min_* and *N_nitri_* in the rhizosphere of shaded offspring ramets were markedly increased by clonal integration (Fig. 5a,b).

### Correlations between the PLFAs concentrations of different microbial groups and soil properties or microbial processes

Soil properties (such as DOC, DON, TOC, NH_4_^+^, NO_3_^−^) were positively correlated to the PLFAs concentrations of most microbial groups except for actinomycete (Table 3). Similarly, microbial processes (such as *N_nitri_*, *N_min_*, invertase and urease activities) were positively correlated to the PLFAs concentrations of most microbial groups (Table 3). On the contrary, POXase and PODase activities were negatively correlated to the PLFAs concentrations of most microbial groups (Table 3). In addition, Ba/Fu and G^−^/G^+^ ratios were not significantly correlated with any of the measured soil properties and microbial processes (Table 3).

**Table 3:** Correlations between concentrations or ratios of soil microbial groups PLFAs and soil properties or microbial processes in the rhizosphere of shaded offspring ramets.

Soil Microbialgroups	Soil properties Microbial processes													
	DOC	DON	TOC	TN	pH	moisture	NH_4_^+^	NO_3_^−^	*N_min_*	*N_nitri_*	Invertase	Urease	POXase	PODase
TP	0.87*	0.89*	0.83*	−0.63	0.56	−0.48	0.86*	0.88*	0.95**	0.88*	0.89*	0.90*	−0.65	−0.83*
Ba	0.90*	0.88*	0.82*	−0.38	0.70	−0.39	0.82*	0.92**	0.94**	0.92**	0.94**	0.94**	−0.82*	−0.92**
Fu	0.86*	0.89*	0.92**	−0.75^+^	0.97**	−0.59	0.77^+^	0.87*	0.82*	0.87*	0.85*	0.72	−0.92**	−0.84*
ECM	0.88*	0.92**	0.98***	−0.64	0.96**	−0.73	0.74^+^	0.92*	0.88*	0.92**	0.89**	0.85*	−0.94**	−0.90*
Ac	0.69	0.64	0.54	−0.16	0.42	0.01	0.69	0.70	0.76^+^	0.70	0.75^+^	0.74^+^	−0.57	−0.69
G^+^	0.97**	0.95**	0.88*	−0.49	0.79^+^	−0.57	0.88*	0.98***	0.96**	0.98***	0.98**	0.97**	−0.90*	−0.98**
G^−^	0.84*	0.83*	0.74^+^	−0.48	0.44	−0.59	0.67	0.85*	0.88*	0.85*	0.84*	0.91*	−0.60	−0.83*
Ba/Fu	0.08	−0.03	−0.14	0.55	−0.34	0.18	0.02	0.05	0.17	0.05	0.09	0.30	0.13	−0.10
G^−^/G^+^	−0.43	−0.40	−0.45	0.11	−0.80^+^	0.12	−0.56	−0.45	−0.34	−0.46	−0.47	−0.32	0.74^+^	0.49

Correlation coefficients and significant levels were shown. Significant levels were indicated by *** (*p*<0.001), ** (*p*<0.01), * (*p*<0.05) and ^+^ (*p*<0.1); n = 10. Abbreviations: TP, total PLFAs; Ba, bacteria; Fu, fungi; ECM, ectomycorrhizal fungi; Ac, actinomycete; G^+^, gram-positive bacteria; G^−^, gram-negative bacteria; Ba/Fu, bacteria/fungi; G^−^/G^+^, gram-negative bacteria/gram-positive bacteria; *N_min_*, nitrogen mineralization rate; *N_nitri_*, nitrogen nitrification rate; POXase, phenol oxidase; PODase, peroxidas.

## Discussion

### Changes in C and N availabilities

A major source of labile C inputting to soil is the root exudates [3], [36], [37]. Because girdling blocked the flow of newly formed photosynthates to the roots and to mycorrhizal fungi, it significantly decreased concentration of DOC and *C_mic_* in the rhizosphere or bulk soils [1], [13], [38]. Clonal plants can translocate or share photosynthates from exposed ramets to shaded ramets by clonal integration [39]. So, effects of clonal integration on labile C may be similar with those reported in previous girdling experiments. Root exudation is associated with increased N availability in the rhizosphere [14]. DON and *N_mic_* were decreased in the girdled plots [38]. The decrease in DOC and DON in girdled plots was probably caused by the decrease in root exudation because a substantial portion of DOC and DON originated from photosynthates [14]. In our study, the increase of belowground carbon allocation caused by clonal integration may improve C and N availability in the rhizosphere of shaded offspring ramets.

Increased dissolved inorganic N concentrations in response to girdling have been found in other studies [14], [40]. On the contrary, we suspect that clonal integration, by increasing the supply of available C to microorganisms, may stimulate N mineralization and nitrificantion in the rhizosphere of shaded offspring ramets. Alternatively, effects of available C supply on soil dissolved inorganic N concentrations may depend on the species-specific. Compared with NH_4_^+^-N, NO_3_^−^-N concentration was lower in the rhizosphere of shaded offspring ramets, regardless of stolon connection or severing (Table 2). This is most likely due to the high mobility of NO_3_^−^ in soil [40].

### Changes in microbial biomass and community composition

DOC is an available C source for microbes and affects their abundance, composition and activity [41]. A larger portion of easily assimilable C was derived from photosynthates produced by plants and shaped a specific microbial community composition [9], [11], [42],[43]. Tree girdling did not affect the total PLFAs concentration and increased the concentration of bacterial PLFAs [38]. In addition, the concentrations of PLFAs for bacterial groups were also related to soil pH [44]. Although no effect of clonal integration on soil pH was observed, the total, bacteria, Gram-positive bacteria and Gram-negative bacteria PLFAs concentrations in the rhizosphere of shaded offspring ramets were significant increased by clonal integration (Table 2; Fig. 3). The similar patterns were observed in another girdling experiment [1].

Specially, clonal integration distinctly increased the PLFAs concentration of ECM fungi (18:2ω6,9) by 72% (Fig. 3g). This was consistent with a previous girdling experiment [13]. Our results further confirm that the dramatic increase in fungi caused by clonal integration is associated with a increase in C supply and that fungi depend to a much higher degree on belowground C allocation [12].

The increase of total fungal biomass caused by clonal integration was mainly related to the increase of ECM fungal biomass [38], [41]. Compared to Gram-positive bacterial PLFAs, the concentrations of Gram-negative bacterial PLFAs were higher in the rhizosphere of shaded offspring ramets, regardless of stolon connection or severing (Fig. 3e,f). This is consistent with the suggestion that Gram-negative bacteria are generally favored by the labile C substrates released by rhizosdeposition and more frequent in the rhizosphere [1]. The effects of clonal integration on actinomycetes were not observed (Fig. 3d). A possible explanation is that actinomycetes are less stimulated in the rhizosphere [45]. Association relationships between different microbial groups (bacteria, Gram-negative bacteria, Gram-positive bacteria, fungi and ECM) and DOC or TOC concentrations may imply their strong dependence (Table 3). We tentatively conclude that the effects of clonal integration on soil C and N availability lead to substantial changes in microbial biomass and community composition in the rhizosphere of shaded offspring ramets.

### Changes in microbial processes

Soil enzyme activities can be used as potential indicators of nutrient cycling processes. Invertase catalyzes hydrolytic processes of SOM [44]. Urease is generally produced by bacteria, filamentous fungi and yeasts, thus enhancing N mineralization [46], [47]. Plant root exudates may provide a constant energy supply, thereby creating optimal conditions for SOM degraders [48]. The increased microbial biomass caused by enhancing root exudation could increase extracellular enzymes activities and the release of N from SOM [49], [50]. The suggestions were further supported by the positive correlations between invertase or urease activities and most soil microbial groups in the rhizosphere of shaded offspring ramets (Table 3).

Phenoloxidase (POXase) and peroxidase (PODase) are the lignolytic enzyme involved in the degradation of recalcitrant SOM (e.g. lignin) [51]. POXase and PODase are generally produced by slow-growing specialist decomposers (e.g. saprotrophic fungi) [52], [53]. Competition between microbial groups could also have been responsible for the shift of enzyme activities. Mycorrhizal fungi are known to dominate the rooted soil layers as a result of a competitive advantage gained through access to root C, whereas saprotrophic fungi are thought to be more competitive in the litter layer [53], [54]. Clonal integration greatly increased the abundance of mycorrhizal fungi, thereby possibly also giving saprotrophic fungi a competitive disadvantage in the rooting zone. The suggestions were further confirmed by the negative correlations between POXase or PODase activities and most soil microbial groups in the rhizosphere of shaded offspring ramets (Table 3). The similar patterns were observed in a previous girdling experiment [1].

Dissolved organic matter (e.g. DOC, DON) was considered to influence soil microbial processes, such as soil respiration/C mineralization [55], [56] and N mineralization [57], [58]. N mineralization and nitrification were regulated by a variety of heterotrophic bacteria and fungi via using labile C source or SOM [40]. N mineralization and nitrification rates were strongly increased by clonal integration in the rhizosphere of shaded offspring ramets (Fig. 5). Positive correlations between N mineralization or nitrification rates and most microbial groups were observed in the rhizosphere of shaded offspring ramets (Table 3). The similar patterns were found in a previous girdling experiment [1]. Notably, *N_nitri_* tended to be much lower than *N_min_* in the rhizosphere of shaded offspring ramets, regardless of stolon connection or severing (Fig. 5). This could be explained by the fact that NO_3_^−^ was derived from the oxidizing of NH_4_^+^ (i.e. nitrification) by chemoautotrophic bacteria or heterotrophic microorganisms [59], [60]. In addition, NO_3_^−^ was water soluble and seldom present in detectable amounts [61].

Because of a high availability of easily assimilable carbon and nutrients, microbial community composition were modified by clonal integration in the rhizosphere of shaded offspring ramets. Invertase and urease activitties, N mineralization and nitrification rates were enhanced by clonal integration in the rhizosphere of shaded offspring ramets. So, clonal integration may facilitate N assimilation and uptake in the rhizosphere of shaded offspring ramets. A field study investigated the effects of clonal integration on nutrient recycling of the Serengeti grassland communities [62]. Our experiment provides insights into the mechanism of nutrient recycling mediated by clonal integration. To allow a robust generalization, however, more experimental studies, especially those conducted in the field, are required.
